# Educational differences in labor market marginalization among mature-aged working men: the contribution of early health behaviors, previous employment histories, and poor mental health

**DOI:** 10.1186/s12889-020-09899-5

**Published:** 2020-11-25

**Authors:** Emelie Thern, Jonas Landberg, Tomas Hemmingsson

**Affiliations:** 1 Department of Public Health Sciences, Stockholm University, 106 91 Stockholm, Sweden; 2 Institute of Environmental Medicine, Karolinska Institute, Stockholm, Sweden; 3 Department of Clinical Neuroscience, Karolinska Institute, Stockholm, Sweden

**Keywords:** Social inequality, Education, Disability pension, Sickness absence, Unemployment, Labor-market marginalization, Mature-aged worker

## Abstract

**Background:**

Social inequalities in labor force participation are well established, but the causes of these inequalities are not fully understood. The present study aims to investigate the association between educational qualification and labor market marginalization (LMM) among mature-aged working men and to examine to what extent the association can be explained by risk factors over the life course.

**Method:**

The study was based on a cohort of men born between 1949 and 1951 who were examined for Swedish military service in 1969/70 and employed in 2000 (*n* = 41,685). Data on educational qualification was obtained in 2000 and information on the outcome of LMM (unemployment, sickness absence, and disability pension) was obtained between 2001 and 2008. Information on early health behaviors, cognitive ability, previous employment histories, and mental health was collected from conscription examinations and nationwide registers.

**Results:**

Evidence of a graded association between years of education and LMM was found. In the crude model, compared to men with the highest level of education men with less than 12 years of schooling had more than a 2.5-fold increased risk of health-related LMM and more than a 1.5-fold increased risk of non-health-related LMM. Risk factors measured across the life course explained a large part of the association between education and health-related LMM (33–61%) and non-health-related LMM (13–58%).

**Conclusions:**

Educational differences remained regarding LMM among mature-aged workers, even after considering several important risk factors measured across the life course. Previous health problems and disrupted employment histories explained the largest part of the associations.

## Background

Labor market marginalization (LMM), i.e. being more or less distant from the labor force due to sickness absence, unemployment or disability pension, among mature-aged workers (50 years or older) is becoming a notable public health and societal challenge, given that the majority of countries are becoming increasingly reliant upon the aging workforce [1]. Although Sweden has one of the highest participation rates of matured-aged workers in the labor force compared to other European countries, almost 20% of the adults (55 to 64 years) were not active in the labor force in 2018 [2]. Mature-aged workers are vulnerable in the labor market as they might be forced to leave the labor force early due to health reasons or job loss; resulting in lower income, well-being, and life satisfaction [3–5]. Evidence suggests that individuals experiencing long-term sickness absence or job loss after the age of 45 face great difficulty returning to work or securing new adequate employment [6, 7]; consequently, LMM in this age group could be a strong risk factor for early permanent retirement. Furthermore, although the existence of social inequalities in labor force participation especially in older ages is well established [8–16], the causes of these inequalities are not fully understood. Thus, there is a need for an enhanced understanding of risk factors across the life course that could explain the social inequalities in labour force participation among mature-aged workers. Subsequently, to be able to target initiatives for sustaining employment up to and beyond retirement ages for the whole population, as this is vital for economic growth and sustainability [1].

The source of social inequalities in labor force participation could be differential exposure to various risk factors of LMM, such as an unequal distribution of poor health, lifestyle factors, and work-related factors [10–19]. The main body of previous research has focused on contemporary health, lifestyle habits, and work-related factors (i.e. heavy workload and poor psychosocial environment), and found that these factors do not sufficiently explain the social inequalities in labor force participation [10–19]. Only a few studies have investigated the contribution of factors measured before labor market entry [16, 18, 19], which could be of importance as adolescence is a sensitive period during the life course when many health behaviors are established that could influence health later in life [20, 21]. Previous research suggests that factors measured in late adolescence, especially IQ, explained a large part of the association between education and disability pension [16, 18, 19]. Furthermore, there has been less focus in the current literature on the risk factor of disrupted work histories over an extended period, which could be of importance as individuals with lower socioeconomic position (SEP) generally experience more sickness absence and unemployment during working life, and these factors appear to be major risk factors of later LMM [17, 22–25].

Thus, to get a better understanding of the underlying causes of the existing social inequalities in labor market participation, the present study aims to investigate the association between educational qualification and LMM among mature-aged working men and to examine to what extent the association can be explained by risk factors over the life course. For this purpose, we will use high-quality nation-wide registers and follow a large cohort of men who were enlisted in the Swedish military service in 1969/70.

## Method

### Study population

The study base included all men born between 1949 and 1951 who were examined for military service in 1969/1970 (*n* = 49,321) in Sweden. During this time military service was obligatory, by law, for all males aged 18–20 years. Only individuals with a severe handicap or congenital disorders were exempted from conscription, which was about 2 to 3% of the general population.

**Fig. 1:**
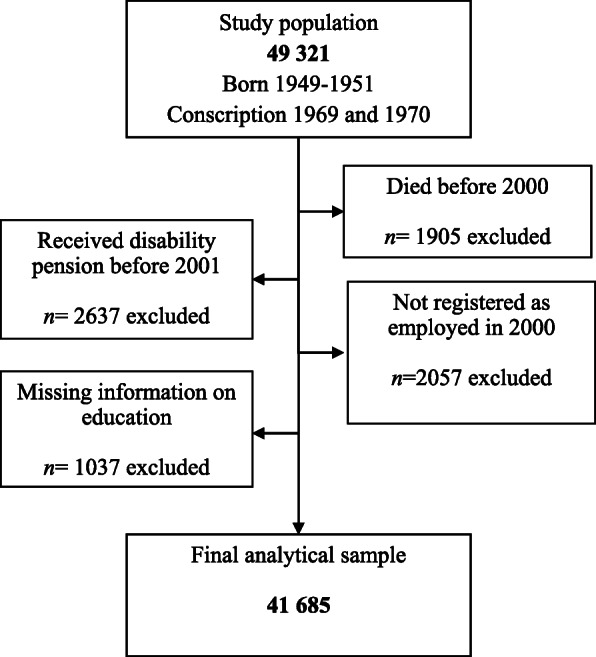
Flow chart describing the selection process of the participants

The study population for the current study included all men still alive and employed in 2000. Individuals that received disability pension before 2001 were excluded, as well as individuals with missing information on the level of education. Excluded participants had lower levels of educational qualification, lower IQ, more health risk behaviors during later adolescence, more disrupted work histories across their working life, and worse health during late adolescence, as well as later in life compared to the included participants (Table S1). The final analytical sample consisted of 41,685 men (Fig. 1).

### Exposure: educational qualification

Information on the highest level of educational attainment, as a measure of SEP in middle age, was collected from the Longitudinal Register of Education and Labour Market Statistics (LISA) in 2000. The LISA-register was established in 1990, updated yearly by Statistics Sweden, and contains information on various factors related to education, income, and the labor market for all citizens from the age of 16 years [26]. The exposure variable of the highest level of education obtained was categorized into five separate groups based on the number of years in education: ≤ 9 years (primary), 10–11 years (2 years of upper secondary school), 12 years (3 years of upper secondary), 13–14 years (2 years of university), and ≥ 15 years (3 or more years of university), with the last group serving as the reference category.

### Outcome: labor market marginalization

In line with previous research, the outcome of LMM was defined using three different measures: disability pension, long-term sickness absence, and long-term unemployment [27]. Information on the outcome was collected from the LISA-register from 1 January 2001 when the participants were 51–53 years old to 31 December 2008 when the participants were 57–59 years old.

In Sweden, disability pension is granted to all citizens (irrespective of employment status) between the age of 19–64, who have a medically confirmed disease or injury that permanently impairs their work capacity by at least 25% [28]. In previous research, long-term sickness absence has been defined as receiving sickness benefits from the Swedish Social Insurance Agency for 90 days or more annually [27]. As we did not have available information on the number of days the individual was home on sick leave, an individual was considered being on long-term sickness absence if at least 25% of their yearly total income came from sickness benefits paid by the Swedish Social Insurance Agency. In line with previous research, long-term unemployment was defined as being registered as unemployed for at least 180 days in 1 year [27].

For the analyses, long-term sickness absence and disability pension were combined as one outcome, since sickness absence is a major risk factor for disability pension in this age-group [17, 23, 24] and 90% of the individuals in the current sample who were granted disability pension during follow-up had received sickness benefits just before being granted disability pension. Thus, the two outcomes were health-related LMM (i.e. long-term sickness absence or disability pension) and non-health-related LMM (i.e. long-term unemployment).

### Covariates

The selection and inclusion of individual- and family-level covariates were based on previous research [10, 16, 27]. Information on childhood SEP was obtained from the Swedish National Population and Housing Census 1960 when the participants were 9–11 years old. Childhood SEP was defined using the fathers’ occupational class, which was categorized into seven groups according to the Swedish socioeconomic classification of occupations: (i) unskilled workers, (ii) skilled workers, (iii) low-level non-manual employees, (iv) intermediate non-manual employees, (v) high-level non-manual employees, (vi) farmers, and (vii) those not classified into a socioeconomic group [29].

The Swedish military service conscription examinations consist of a standard medical assessment of physical and mental health, as well as two survey questionnaires. The questionnaires used at the conscription examinations have not been previously published, however, several previous studies have used information from the conscription examination as this data is available to researchers [16, 30]. The following information was obtained from the conscription examination; IQ, smoking, alcohol consumption, body mass index (BMI), emotional control, and psychiatric and musculoskeletal diagnoses. The IQ test administered to the enlistees was based on four subtests designed to capture logical, spatial, verbal, and technical abilities [31]. From the four subtests, a global IQ score was calculated and standardized to give a Gaussian-distributed score between 1 and 9, with higher values indicating greater intellectual capacity. Health behaviors (i.e. smoking, alcohol, and BMI) were defined in line with previous research [16]. Participants smoking five cigarettes per day or more were considered as smokers. Risky use of alcohol was defined as consuming at least 250 g of 100% alcohol per week, using alcohol as an eye-opener, have been apprehended for drunkenness, or reported being drunk rather often or often. Height and weight were used to calculate BMI (weight/height^2^). Participants with a BMI of ≥25 at conscription were considered overweight. Low emotional control (rated by a psychologist) was defined as having low-stress tolerance and/or anxiety, reduced functioning due to psychosomatic symptoms, and uncontrollable nervousness, anxiousness, or aggression. All enlistees were examined by a physician and if considered necessary a psychiatrist. Psychiatric and musculoskeletal diagnoses were defined according to the International Classification of Diseases version 8 (ICD-8); codes 290–315 and 710–738, respectively.

Disrupted work histories were defined using three different measures of unemployment: youth unemployment, unemployed in young adulthood, and unemployed in middle adulthood. Youth unemployment was defined as experiencing more than 3 months of unemployment before the age of 18 and obtained from the conscription examination. Adapted from previous research [30], information from the Swedish Income and Tax Register on receiving unemployment benefits (Unemployment Insurance Funds) or unemployment assistance (Social Insurance Agency) was obtained between 1974 and 1991. Individuals receiving any benefit or assistance for at least 4 out of the 15 years were classified as been unemployed in young adulthood. Information on long-term unemployment between 1992 and 2000 was obtained from the LISA-register using the same definition as for the outcome variable. Participants were considered having experienced unemployment in middle adulthood if this was reported at least once between 1992 and 2000.

Two measures of health-related factors were also included in the model; long-term sickness absence and receiving care for a psychiatric diagnosis. Information on long-term sickness absence was collected from 1990 to 2000 from the LISA register using the same definition as for the outcome, individuals were considered as been on long-term sickness absence if this was reported once between 1990 and 2000. Information on psychiatric diagnoses was obtained from the National Hospital Discharge Register between 1973 and 2000 (i.e. between the ages of 22–24 years and 50–52 years). Psychiatric diagnoses were defined by the following discharge diagnostic codes: ICD-8 and ICD-9: 290–315; ICD-10: F00-F99.

### Statistical analysis

Pearson’s chi-square tests (χ^2^) were used to test for differences in baseline characteristics of the study population. The association between level of education and later LMM was estimated by Cox proportional hazard models to obtain hazard ratios (HR) with 95% confidence intervals (CI). The proportional hazards assumptions were tested using Schoenfeld residuals and found not to be violated (all *p*-values ≥0.05). Information on the outcomes was obtained from registers that are updated annually; consequently, there was no exact date available for when the disability pension, sickness benefits, or unemployment benefits was received. Therefore, the day and month of the outcome were set on the 2nd of July as this is the middle of the year. Person-time was calculated from 1 January 2001 until the date of labor market marginalization, date of death, or until the end of follow-up (31 December 2008), whichever came first. The end of follow-up was set in 2008 due to register constraints.

Covariates were included individually as well as grouped according to a chronological timeline, we first included childhood SEP, then variables measured at conscription, then variables related to prior work histories, and finally health-related factors measured across the life course. In the final model, all variables were included simultaneously. Multicollinearity among the predictors was examined using the variance inflation factor (VIF); as all VIF values were below five, we determined that the potential issue of multicollinearity did not merit further investigation [32]. The percentage of HR reduction was calculated as ((HR_crude_-HR_adjusted_)/(HR_crude_-1)) *100.

Additional analyses were conducted for the outcome of long-term unemployment, where individuals experiencing long-term sickness absence before the unemployment spell were excluded (*n* = 549), as long-term sickness absence could be a risk factor for later unemployment [17]. Furthermore, competing risk analyses in accordance with the method of Fine and Gray (1999) were conducted for both outcomes, including the date of death as a competing event, since the risk of death was not randomly distributed between the exposure groups. Missing values on covariates were coded as separate categories, as similar results were obtained when individuals with missing information on covariates were excluded (i.e. complete case analyses) (Table S2 and S3). All analyses were performed using Stata Statistical Software: Release 15.

## Results

### Baseline characteristics

The baseline characteristics of the study population, stratified by the highest level of educational qualification, can be found in Table 1. Those with lower levels of education had generally lower childhood SEP, lower IQ, more unhealthy habits during later adolescence, lower emotional control, and worse mental health in adolescence as well as later in life compared to individuals with higher levels of education. Individuals with higher levels of education had generally less disrupted work histories and fewer health problems compared to individuals with lower levels of education.

**Table 1:** Baseline characteristics of study population, stratified by years of education

Total	≥15 n(%)	13–14 n(%)	12 n(%)	10–11 n(%)	≤9 n(%)	*p*-value
	7327 (15.6)	6155 (14.8)	6664 (16.0)	11,915 (28.6)	9624 (23.1)	
Childhood SEP^a^						
Unskilled worker	1311 (17.9)	1594 (25.9)	2023 (30.4)	4570 (38.4)	4144 (43.1)	< 0.001
Skilled worker	1039 (14.2)	1265 (20.6)	1481 (22.2)	2998 (25.2)	2162 (22.5)	
Low-level non-manual employee	1083 (14.8)	809 (13.1)	781 (11.7)	1019 (8.6)	586 (6.1)	
Intermediate non-manual employee	2246 (30.7)	1366 (22.2)	1253 (18.8)	1415 (11.9)	739 (7.7)	
High-level non-manual employee	1054 (14.4)	347 (5.6)	361 (5.4)	261 (2.2)	170 (1.8)	
Farmer	473 (6.5)	668 (10.9)	661 (9.9)	1362 (11.4)	1600 (16.6)	
Not classified	121 (1.7)	106 (1.7)	104 (1.6)	290 (2.4)	223 (2.3)	
IQ^b^						
High [7–9]	5000 (68.2)	3369 (54.7)	2509 (37.7)	2058 (17.3)	867 (9.0)	< 0.001
Medium [4–6]	2189 (29.9)	2559 (41.6)	3526 (52.9)	7233 (60.7)	5133 (53.3)	
Low [1–3]	130 (1.8)	220 (3.6)	622 (9.3)	2605 (21.9)	3610 (37.5)	
Missing	8 (0.1)	7 (0.1)	7 (0.1)	19 (0.2)	14 (0.2)	
Health behaviors^b^						
Smoking ≥5 cigarettes/day	2086 (28.5)	2167 (35.2)	2929 (44.0)	6183 (51.9)	5347 (55.6)	< 0.001
Risky use of alcohol	1027 (14.0)	909 (14.8)	1214 (18.2)	2775 (23.3)	2343 (24.4)	< 0.001
BMI ≥25	263 (3.6)	318 (5.2)	393 (5.9)	841 (7.1)	820 (8.5)	< 0.001
Low emotional control^b^	1753 (23.9)	1428 (23.2)	1616 (24.3)	3539 (29.7)	3294 (34.2)	< 0.001
Psychiatric diagnosis^b^	567 (7.7)	426 (6.9)	532 (8.0)	1360 (11.4)	1336 (13.9)	< 0.001
Musculoskeletal diagnosis^b^	1229 (16.8)	988 (16.1)	1013 (15.2)	1981 (16.6)	1698 (17.6)	0.001
Employment histories						
Youth unemployment^b^	205 (2.8)	261 (4.2)	402 (6.0)	1795 (15.1)	1898 (19.7)	< 0.001
Unemployed in young adulthood^c^	210 (2.9)	241 (3.9)	231 (3.5)	992 (8.3)	484 (5.0)	
Unemployed in middle adulthood^d^	576 (7.9)	642 (10.4)	886 (13.3)	2341 (19.7)	1423 (14.8)	< 0.001
Health factors						
Long-term sick leave^e^	447 (6.5)	607 (9.9)	962 (14.4)	2244 (18.8)	1918 (19.9)	< 0.001
Inpatient-care psychiatric diagnosis^f^	174 (2.4)	185 (3.0)	227 (3.4)	691 (5.8)	537 (5.6)	< 0.001
Outcome^g^						
Health-related LMM	670 (9.1)	830 (13.5)	1130 (17.0)	2726 (22.9)	2357 (24.5)	< 0.001
Non-health-related LMM	339 (5.5)	448 (7.3)	564 (8.5)	1295 (10.9)	820 (8.5)	< 0.001

*SEP* Socioeconomic position, *BMI* Body mass index

^a^Measured in 1960

^b^Measured during conscription in 1969

^c^Measured from 1974 to 1991

^d^Measured from 1992 to 2000

^e^Measured from 1990 to 2000

^f^Measured from 1973 to 2000

^g^Measured between 2001 and 2008

During follow-up, a total of 7713 (18.5%) individuals were on long-term sick leave or received disability pension and 3526 (8.5%) experienced long-term unemployment (Table 1). Follow-up time was on average 7 years for health-related LMM and 7.5 years for non-health-related LMM. During follow-up, a total of 1031individuals died, of which 302 (3.1%) had the lowest level of education and 126 (1.7%) had the highest level of education. The separate and unadjusted effects of all risk factors on health-related and non-health-related LMM are shown in Table 2. All risk factors included in the current study were positively associated with both outcomes, except for childhood SEP, BMI, and musculoskeletal diagnosis with non-health-related LMM.

**Table 2:** Unadjusted HRs and 95% CIs on the association between each potential risk factor and health-related LMM and non-health-related LMM (2001–2008)

	Health-related LMM (*n* = 7713) HR (95%CI)	Non-health-related LMM (*n* = 3526) HR (95%CI)
Childhood SEP		
Unskilled worker	1.65 (1.46, 1.87)	1.09 (0.93, 1.28)
Skilled worker	1.60 (1.41, 1.81)	1.07 (0.91, 1.26)
Low-level non-manual employee	1.23 (1.07, 1.41)	0.98 (0.82, 1.17)
Intermediate non-manual employee	1.15 (1.01, 1.31)	1.07 (0.91, 1.26)
High-level non-manual employee (ref)	1.00	1.00
Farmer	1.71 (1.49, 1.95)	0.73 (0.61, 0.88)
IQ		
High [7–9] (ref)	1.00	1.00
Medium [4–6]	1.67 (1.58, 1.77)	1.25 (1.15, 1.35)
Low [1–3]	2.32 (2.17, 2.48)	1.78 (1.62, 1.96)
Health behaviors		
Smoking	1.43 (1.37, 1.50)	1.31 (1.23, 1.41)
Risky use of alcohol	1.30 (1.23, 1.37)	1.31 (1.21, 1.41)
BMI ≥25	1.31 (1.20, 1.42)	1.06 (0.93, 1.21)
Low emotional control	1.47 (1.40, 1.54)	1.37 (1.28, 1.47)
Psychiatric diagnosis	1.64 (1.54, 1.74)	1.56 (1.42, 1.72)
Musculoskeletal diagnosis	1.23 (1.16, 1.30)	1.03 (0.94, 1.12)
Employment histories		
Youth unemployment	1.49 (1.40, 1.59)	1.58 (1.44, 1.73)
Unemployed in young adulthood	1.95 (1.80, 2.11)	3.08 (2.80, 3.40)
Unemployed in middle adulthood	1.84 (1.74, 1.94)	5.19 (4.85, 5.54)
Health factors		
Long-term sick leave	4.97 (4.75, 5.21)	1.76 (1.63, 1.91)
Inpatient-care psychiatric diagnosis	2.90 (2.70, 3.13)	2.40 (2.14, 2.69)

*LMM* Labor market marginalization, *BMI* Body mass index

### Education and health-related LMM

As seen in Table 3, evidence of a graded association between years of education and health-related LMM was found. Individuals with a lower level of education had more than a 2.50-fold increased risk compared to individuals with the highest level of education in the crude analysis. Risk factors measured in childhood and during adolescence, as well as health-related factors, appeared to contribute to the largest reduction of the hazard ratios in the multivariable analysis. The largest attenuation of the association was found among individuals with lower levels of education. After including all potential risk factors, the increased risk found among all individuals with less than 15 years of education was reduced by 33 to 61%.

**Table 3:** Crude and adjusted hazard ratios (HRs) with 95% confidence intervals (CIs) for the association between level of education (years) and health-related labor market marginalization among mature aged workers

Adjustments	≥15 HR (95%CI)	13–14 HR (95%CI)	12 HR (95%CI)	10–11 HR (95%CI)	≤9 HR (95%CI)
**Crude**	1.00	1.51 (1.36, 1.67)	1.93 (1.75, 2.12)	2.70 (2.48, 2.93)	2.90 (2.66, 3.16)
Childhood SEP (1960)	1.00	1.47 (1.33, 1.63)	1.88 (1.70, 2.07)	2.58 (2.36, 2.81)	2.75 (2.51, 3.00)
IQ (1969)	1.00	1.45 (1.31, 1.61)	1.75 (1.59, 1.93)	2.26 (2.07, 2.47)	2.31 (2.10, 2.53)
Health behaviors (1969)	1.00	1.49 (1.34, 1.64)	1.85 (1.68, 2.04)	2.53 (2.32, 2.75)	2.68 (2.46, 2.93)
Low emotional control (1969)	1.00	1.52 (1.37, 1.68)	1.93 (1.76, 2.12)	2.65 (2.44, 2.88)	2.80 (2.57, 3.06)
Psychiatric diagnosis (1969)	1.00	1.52 (1.37, 1.68)	1.93 (1.75, 2.12)	2.66 (2.44, 2.89)	2.82 (2.59, 3.07)
Musculoskeletal diagnosis (1969)	1.00	1.51 (1.37, 1.67)	1.94 (1.76, 2.13)	2.70 (2.48, 2.94)	2.90 (2.66, 3.16)
**Adjusted for all above**	1.00	1.42 (1.28, 1.58)	1.69 (1.53, 1.86)	2.09 (1.91, 2.30)	2.07 (1.88, 2.28)
**% reduction of HR**		17%	26%	35%	44%
Youth unemployment (1969)	1.00	1.51 (1.36, 1.67)	1.92 (1.74, 2.11)	2.63 (2.41, 2.86)	2.80 (2.57, 3.05)
Unemployed in young adulthood (1974–1991)	1.00	1.50 (1.35, 1.66)	1.92 (1.75, 2.12)	2.60 (2.39, 2.83)	2.86 (2.62, 3.11)
Unemployed in middle adulthood (1992–2000)	1.00	1.49 (1.34, 1.65)	1.87 (1.70, 2.06)	2.52 (2.31, 2.74)	2.79 (2.56, 3.04)
**Adjusted for all unemployment variables**	1.00	1.48 (1.34, 1.64)	1.86 (1.69, 2.05)	2.43 (2.23, 2.64)	2.70 (2.48, 2.95)
**% reduction of HR**		6%	7%	16%	10%
Long-term sick leave (1990–2000)	1.00	1.41 (1.27, 1.56)	1.62 (1.47, 1.78)	2.10 (1.93, 2.28)	2.21 (2.02, 2.41)
Mental diagnosis (1973–2000)	1.00	1.50 (1.35, 1.66)	1.91 (1.73, 2.10)	2.59 (2.38, 2.82)	2.78 (2.55, 3.03)
**Adjusted for all health-related variables**	1.00	1.41 (1.27, 1.56)	1.62 (1.47, 1.78)	2.06 (1.90, 2.25)	2.19 (2.00, 2.38)
**% reduction of HR**		19%	33%	37%	38%
Full model	1.00	1.34 (1.21, 1.48)	1.46 (1.32, 1.62)	1.69 (1.54, 1.85)	1.75 (1.59, 1.93)
**% reduction of HR**		33%	50%	59%	61%

*SEP* Socioeconomic position

### Education and non-health-related LMM

Table 4 shows the association between years of education and unemployment. Individuals with the lowest level of education had a slightly lower risk of long-term unemployment compared to the other groups in the multivariable analyses. Risk factors measured during childhood and late adolescence had a negligible effect on the risk estimates especially among individuals with at least 10 years of education. The largest attenuation of the risk estimates was found after including measures of disrupted work histories for all groups. Altogether, the risk factors attenuated the association between level of education and long-term unemployment by 13 to 58%.

**Table 4:** Crude and adjusted hazard ratios (HRs) with 95% confidence intervals (CIs) for the association between level of education (years) and non-health-related labor market marginalization among mature aged workers

Adjustments	≥15 HR (95%CI)	13–14 HR (95%CI)	12 HR (95%CI)	10–11 HR (95%CI)	≤9 HR (95%CI)
**Crude**	1.00	1.35 (1.18, 1.55)	1.58 (1.39, 1.80)	2.06 (1.84, 2.31)	1.60 (1.41, 1.80)
Childhood SEP (1960)	1.00	1.41 (1.23, 1.61)	1.65 (1.45, 1.88)	2.19 (1.95, 2.46)	1.74 (1.54, 1.97)
IQ (1969)	1.00	1.32 (1.16, 1.52)	1.49 (1.31, 1.70)	1.82 (1.61, 2.05)	1.32 (1.16, 1.51)
Health behaviors (1969)	1.00	1.34 (1.17, 1.53)	1.53 (1.34, 1.74)	1.94 (1.74, 2.18)	1.49 (1.32, 1.68)
Low emotional control (1969)	1.00	1.36 (1.18, 1.55)	1.58 (1.39, 1.79)	2.03 (1.81, 2.27)	1.55 (1.37, 1.74)
Psychiatric diagnosis (1969)	1.00	1.36 (1.18, 1.55)	1.58 (1.39, 1.79)	2.03 (1.81, 2.27)	1.55 (1.38, 1.75)
Musculoskeletal diagnosis (1969)	1.00	1.35 (1.18, 1.55)	1.58 (1.39, 1.80)	2.06 (1.84, 2.31)	1.60 (1.42, 1.80)
**Adjusted for all above**	1.00	1.38 (1.21, 1.58)	1.54 (1.35, 1.76)	1.86 (1.64, 2.11)	1.37 (1.19, 1.57)
**% reduction of HR**		+ 9%	7%	18%	39%
Youth unemployment (1969)	1.00	1.34 (1.17, 1.54)	1.56 (1.37, 1.77)	1.96 (1.74, 2.19)	1.49 (1.32, 1.68)
Unemployed in young adulthood (1974–1991)	1.00	1.33 (1.16, 1.52)	1.57 (1.38, 1.78)	1.90 (1.70, 2.13)	1.54 (1.37, 1.74)
Unemployed in middle adulthood (1992–2000)	1.00	1.27 (1.11, 1.45)	1.38 (1.21, 1.56)	1.56 (1.39, 1.75)	1.34 (1.19, 1.51)
**Adjusted for all unemployment variables**	1.00	1.26 (1.10, 1.44)	1.37 (1.20, 1.57)	1.46 (1.30, 1.64)	1.28 (1.13, 1.44)
**% reduction of HR**		27%	36%	56%	53%
Long-term sick leave (1990–2000)	1.00	1.33 (1.16, 1.52)	1.51 (1.33, 1.71)	1.92 (1.71, 2.15)	1.47 (1.31, 1.66)
Mental diagnosis (1973–2000)	1.00	1.34 (1.17, 1.54)	1.56 (1.37, 1.78)	1.99 (1.77, 2.22)	1.54 (1.37, 1.74)
**Adjusted for all health-related variables**	1.00	1.32 (1.16, 1.51)	1.50 (1.32, 1.71)	1.88 (1.68, 2.10)	1.45 (1.28, 1.64)
**% reduction of HR**		8%	13%	17%	25%
Full model	1.00	1.30 (1.14, 1.49)	1.39 (1.21, 1.59)	1.46 (1.28, 1.65)	1.25 (1.09, 1.43)
**% reduction of HR**		13%	33%	57%	58%

*SEP* Socioeconomic position

### Additional analyses

Results from the additional analyses, when individuals with prior health-related LMM where excluded, demonstrated a similarly increased risk of non-health-related LMM among individuals with lower levels of education as in the main analysis (Table S4). Similar results were obtained as in the main analyses when including the date of death as a competing event (Table S5 and S6).

## Discussion

The results of this study suggest that educational differences in LMM among mature-aged men remain after considering several risk factors measured across the life course. Generally, disrupted employment histories and previous health problems explain the largest part of the association between education and LMM among mature-aged workers.

These results support and further extend the current literature on the social inequalities in LMM in several ways [8–16]. First, in concordance with earlier studies, we found that risk factors measured across the life-course seems to partly explain the increased risk of LMM among individual with lower educational qualifications [10–19]. Extending previous research on social inequalities in labor market participation three measures of disrupted work histories during the life course were included, which was found to be one of the main risk factors of LMM and explain the largest part of the association between education and LMM. Previous research has found that prior unemployment is a major risk factor of LMM [17, 23–25], but less was known of how prior disrupted employment histories during the life course could explain the social inequalities in LMM. This is of great importance as individuals with fewer years of education reported prior unemployment spells in youth and during adulthood to a greater extent, suggesting that the individuals with lower SEP are more vulnerable on the labor market throughout working life which increases their risk of LMM later in life. In the current study, experiencing unemployment later in life appeared to explain a larger part of the association between education and non-health-related LMM among mature-aged workers compared to youth unemployment and unemployment in young adulthood among all groups. It should, however, be acknowledged that the information regarding prior unemployment during middle adulthood was collected during the 1990s, a period when Sweden was hit by an economic downturn; sickness absence and unemployment drastically increased during this time, from 1.7% in 1990 to 8.3% in 1993 [33]. Consequently, although some groups were hit harder (i.e. women, young adults, and immigrants), the unemployment experience during the economic crises was less influenced by health selection and more widespread to all sectors of the workforce [33]. To reduce this potential issue, we included several measures of unemployment outside of this period, as well as setting a high limit of unemployment days (i.e. unemployed at least 180 days) to define someone as unemployed during this period. Individuals excluded from the analytical sample reported lower levels of educational qualification and more disrupted work histories compared to the included individuals. Consequently, prior unemployment spells could potentially explain a larger part of the social inequalities in labor force participation.

Previous research has shown that prior sickness absence is a strong risk factor for later sickness absence and disability pensions [17, 23]. Extending these findings, our results showed that differences in prior sickness absence during the life course explained a large part of the association between educational qualification and health-related LMM. Furthermore, our results suggest that receiving a psychiatric diagnosis, in late adolescence or later in life, explained very little of the association between educational qualification and LMM, which is in line with similar previous research [16]. Other research, where the focus has not been on educational differences, has found that childhood and adult psychological health problems and worse general health were positively associated with unemployment, permanent sickness, and early exit later in life [34, 35]. Workers, and especially mature-aged workers, are a selected group with better health compared to same-aged individuals outside of the labor force. This became evident in the exclusion analysis, whereas mental health problems were more prevalent among the excluded participants compared to the included participants (4.4% compared to 27.8%). In Sweden, the two most common underlying diagnoses for sickness absence or being granted disability pension are musculoskeletal diagnoses and mental disorders [36]. Unfortunately, we did not have access to information on the underlying diagnoses of the sickness absence or disability pension in the current study; thus, we are unable to examine the primary reason for the health-related LMM.

Previous research on social inequalities in disability pension has found that factors measured in late adolescence, especially IQ, explained the largest part of the association [16, 18, 19]. This was also found in the current study, when long-term sickness absence was also included in the outcome, among the men with the lowest level of education and in relation to health-related LMM. IQ and educational qualification appear to be strongly linked as previous research suggest that low IQ is an important predictor for unsuccessful educational achievement at age 30 [37]. Even though IQ and education are highly correlated they can still act as moderators for each other in relation to health outcomes [38, 39]; for example, cognitive ability is an independent predictor of long-term sickness absence [40]. We found that health behaviors measured during adolescence contributed little in explaining the educational differences found concerning LMM. Previous research has found that health behaviors measured during adolescence contribute little to explaining the socioeconomic differences in all-cause disability pension, though they explain a large part of the differences concerning disability pension due to an alcohol disorder [16, 19]. Even though adolescence can be considered a sensitive period where most health behaviors are initiated and established, many of the health behaviors included in the study could have changed [21], especially considering the major changes that have occurred in population lifestyles [41]. Subsequently, including a measure of current health behaviors might have resulted in larger attenuations of the risk estimates among the individuals with lower levels of education, which has been seen in previous research on social inequalities in sickness absence where current obesity and smoking status explained a large part of the association [10]. On the other hand, previous research on smoking trajectories from adolescence to later adulthood has found a positive association between smoking during adolescence and later unemployment irrespective of later smoking status [42].

It should also be recognized that the Swedish social insurance system went through some changes during the follow-up period of the current study, which could have influenced the results. The disability pension system was moved from the pension system to the social security system in 2003, following a name change to disability benefit to reinforce the perception that receiving this benefit was not a permanent exit from the labor market, which it had previously been [28]. The calculation of payments was also changed but the eligibility criterion remained the same. Furthermore, the Swedish government wanted to halve the number of individuals being granted sickness compensation and disability benefits by the year 2008, which resulted in a drastic decrease in the number of newly granted disability beneficiaries [43]. Previous research suggests that changes in a social system, such as applying a stricter criteria for eligibility of disability pension or increasing the retirement age, often result in a change in the occurrence of using alternative exit routes from the labor market, such as disability pension, unemployment benefits or early retirement, especially among individuals with poor health [12, 44, 45]. Since we included such a wide definition of LMM, we would most likely capture the individuals forced to use an alternative route to disability pension and thus this reform should not have too much influence on the current result.

To achieve the sustainability developmental goals concerning population aging, it has been suggested that governments should gradually increase the official retirement age, which Sweden and many other countries are currently trying to implement [1]. The results of this study and previous research suggest that this might increase the health inequalities among the older populations as opportunities to be able to extend working life might be dependent on SEP [9, 15]. Taking a life-course perspective to gain a better understanding of the determinants and reasons for leaving the labor market early, especially among those with lower levels of education and income, is important in promoting more stable work participation. Around 24% of the current study population experienced LMM at a mature age and only around half returned to the workforce during follow-up (data not shown), suggesting that being marginalized from the workforce after the age of 50 could be a strong risk factor for an early permanent exit from the labor force. Mature aged workers are vulnerable on the labor market and face great difficulty returning to the labor force in case of sickness absence or unemployment; thus, measures need to be taken much earlier to ensure participation in the labor force is made possible for all older workers [6, 7].

### Strengths and limitations

Conducting a register derived study following a large cohort of men with a long-term follow-up is a major strength. Also, linking nation-wide registers to the rich data source of the conscription cohort with self-reported data on several health measures and health behaviors allowed us to include information on several important risk factors across the life course. Unfortunately, risk factors such as health behaviors were only measured in late adolescence and have most likely changed during the life course; thus, these measures are most likely not an accurate representation of health behaviors among mature-aged men. The information from the conscription was not collected retrospectively, minimizing the issue of recall bias. An obvious shortcoming of the conscription cohort is that it only includes men. Previous research has found the association between education and earlier exit from the labor market due to disability pension is quite similar among males and females, though perhaps somewhat steeper for males, even though females are overrepresented among disability beneficiaries [18]. In the current study, we found that disrupted work histories were an important factor for males, which could be of interest to further investigate for females who often have more disrupted work histories due to childcare.

The risk of self-report bias and attrition was decreased by collecting information on the exposure and outcome from high-quality registers. Using the highest level of educational qualification as an indicator of SEP could be a potential limitation, as it might lose discriminatory power with old age; however, due to register constraints, we were unable to include another common indicator of SEP such as occupational class. Concerning the outcome, we did not have an exact date when the individual was marginalized from the labor force. This limitation in the register should not have influenced the main results as we choose a date mid-year to censor individuals in the analysis and there is most likely no seasonal variation in the number of individuals being on sick-leave, unemployed, or granted disability pension. Furthermore, the amount of unemployment, sickness absence, and disability pension benefits received depend on your salary (less than 80% of your salary), but there is an upper limit to how much money one can receive each day. Consequently, high-income earners will be given the maximum benefit allowed regardless of their actual income which means that there could be some misclassification with regards to long-term sick leave as the definition was based on calculating a percentage of sickness benefit received in relation to income.

A strength was the ability to include several measures of previous unemployment spells, as these factors appear to be of importance. However, a limitation is that we were unable to include any information on the work environment with regards to physical workload and psychosocial environment which appear to be important determinates of disability pension and early retirement [13, 46]. Manual workers, who often have lower levels of education, have more adverse physical work environments which increases their risk of early retirement due to health issues.

## Conclusion

Lower levels of educational qualification increased the risk of being marginalized from the workforce after the age of 50 years, even after considering several risk factors measured across the life course. Bearing in mind the ambition to increase the statutory retirement age in many countries, it is important to study determinants of LMM and an early exit from the labor market especially among individuals with low SEP to be able to lengthen labor force participation in the whole population.

## Supplementary Information

**Additional file 1**: **Supplementary Table 1**. Baseline characteristics of the individuals included and excluded in the study population. **Supplementary Table 2**. Complete case analysis excluding 1908 individuals with missing information on covariates, crude and adjusted hazard ratios (HRs) with 95% confidence intervals (CIs) for the association between educational qualification and health-related labor market marginalization among mature-aged workers. **Supplementary Table 3**. Complete case analysis excluding 1908 individuals with missing information on covariates, crude and adjusted hazard ratios (HRs) with 95% confidence intervals (CIs) for the association between educational qualification and non-health-related labor market marginalization among mature-aged workers. **Supplementary Table 4**: Additional analyses excluding 549 individuals that experienced long-term sickness absence prior to long-term unemployment, crude and adjusted hazard ratios (HRs) with 95% confidence intervals (CIs) for the association between educational qualification and non-health-related labor market marginalization among mature aged workers. **Supplementary Table 5**. Competing risk regression for the association between level of education (years) and health-related labor market marginalization among mature aged workers, with death as competing risk. **Supplementary Table 6**. Competing risk regression for the association between level of education (years) and non-health-related labor market marginalization among mature aged workers, with death as competing risk.

## Data Availability

The data that support the findings of this study are available from Statistics Sweden but restrictions apply to the availability of these data, which were used under license for the current study, and so are not publicly available. Data are however available from the authors upon reasonable request and with permission of Statistics Sweden.

## Abbreviations

LMM: Labor Market Marginalization
LISA: Longitudinal Register of Education and Labour Market Statistics
SEP: Socioeconomic position
BMI: Body mass index
