# Supplementary material for: Educational differences in labor market marginalization among mature-aged working men: the contribution of early health behaviors, previous employment histories, and poor mental health
Source: BMC Public Health. 2020 Nov 25;20:1784. doi: 10.1186/s12889-020-09899-5 (PMC7691056; doi:10.1186/s12889-020-09899-5)
Supplement: Supplementary file 1 — Additional file 1: Supplementary Table 1. Baseline characteristics of the individuals included and excluded in the study population. Supplementary Table 2. Complete case analysis excluding 1908 individuals with missing information on covariates, crude and adjusted hazard ratios (HRs) with 95% confidence intervals (CIs) for the association between educational qualification and health-related labor market marginalization among mature-aged workers. Supplementary Table 3. Complete case analysis excluding 1908 individuals with missing information on covariates, crude and adjusted hazard ratios (HRs) with 95% confidence intervals (CIs) for the association between educational qualification and non-health-related labor market marginalization among mature-aged workers. Supplementary Table 4: Additional analyses excluding 549 individuals that experienced long-term sickness absence prior to long-term unemployment, crude and adjusted hazard ratios (HRs) with 95% confidence intervals (CIs) for the association between educational qualification and non-health-related labor market marginalization among mature aged workers. Supplementary Table 5. Competing risk regression for the association between level of education (years) and health-related labor market marginalization among mature aged workers, with death as competing risk. Supplementary Table 6. Competing risk regression for the association between level of education (years) and non-health-related labor market marginalization among mature aged workers, with death as competing risk. [file 12889_2020_9899_MOESM1_ESM.docx]

Supplementary table 1: Baseline characteristics of the individuals included and excluded in the study population

|  | Analytical sample  n(%) | Excluded  n(%) | p-value |
| --- | --- | --- | --- |
| Total | 41 685 (84.5) | 7636 (15.5) |  |
| Childhood SEP^a^  Unskilled worker  Skilled worker  Low-level non-manual employee  Intermediate non-manual employee  High-level non-manual employee  Farmer  Not classified | 13 642 (32.7)  8945 (21.5)  4278 (10.3)  7019 (16.8)  2193 (5.3)  4764 (11.4)  844 (2.0) | 2709 (35.5)  1602 (21.0)  719 (9.4)  1287 (16.9)  391 (5.1)  655 (8.6)  273 (3.6) | <0.001 |
| IQ^b^  High (7-9)  Medium (4-6)  Low (1-3)  Missing | 13 803 (33.1)  20 640 (49.5)  7187 (17.2)  55 (0.1) | 1788 (23.4)  3596 (47.1)  2232 (29.2)  20 (0.3) | <0.001 |
| Health behaviors^b^  Smoking ≥5 cigarettes/day  Risky use of alcohol  BMI ≥25 | 18 712 (44.9)  8268 (19.8)  2635 (6.3) | 4280 (56.1)  2243 (29.4)  600 (7.9) | <0.001  <0.001  <0.001 |
| Low emotional control^b^ | 11 630 (27.9) | 3297 (43.2) | <0.001 |
| Psychiatric diagnosis^b^ | 4221 (10.1) | 1809 (23.7) | <0.001 |
| Musculoskeletal diagnosis^b^ | 6909 (16.6) | 1415 (18.5) | <0.001 |
| Employment histories  Youth unemployment^b^  Unemployed in young adulthood^c^  Unemployed in middle adulthood^d^ | 4560 (10.9)  2158 (5.2)  5868 (14.1) | 1576 (20.6)  909 (11.9)  4845 (63.5) | <0.001  <0.001 |
| Health factors  Long-term sick leave^e^  Inpatient-care psychiatric diagnosis^f^ | 6208 (14.9)  1814 (4.4) | 3346 (43.9)  2126 (27.8) | <0.001 |
| Years of education  ≤9  10-11  12  13-14  ≥15  Missing | 9624 (23.1)  11 915 (28.6)  6664 (16.0)  6155 (14.8)  7327 (17.6)  0 | 1539 (20.2)  1816 (23.8)  625 (8.2)  337 (4.4)  332 (4.4)  2987 (38.1) | <0.001 |

SEP: socioeconomic position, BMI: Body mass index

^a^Measured in 1960

^b^Measured during conscription in 1969

^c^Measured from 1974 to 1991

^d^Measured from 1992 to 2000

^e^Measured from 1990 to 2000

^f^Measured from 1973 to 2000

Supplementary table 2. Complete case analysis excluding 1908 individuals with missing information on covariates, crude and adjusted hazard ratios (HRs) with 95% confidence intervals (CIs) for the association between educational qualification and health-related labor market marginalization among mature-aged workers

| Adjustments | ≥15  HR (95%CI) | 13-14  HR (95%CI) | 12  HR (95%CI) | 10-11  HR (95%CI) | ≤9  HR (95%CI) |
| --- | --- | --- | --- | --- | --- |
| **Crude** | 1.00 | 1.49 (1.34, 1.65) | 1.90 (1.73, 2.10) | 2.64 (2.43, 2.88) | 2.88 (2.64, 3.15) |
| Childhood SEP (1960) | 1.00 | 1.45 (1.31, 1.61) | 1.84 (1.67, 2.04) | 2.51 (2.30, 2.75) | 2.71 (2.47, 2.97) |
| IQ (1969) | 1.00 | 1.43 (1.29, 1.59) | 1.73 (1.56, 1.91) | 2.21 (2.02, 2.42) | 2.29 (2.07, 2.51) |
| Health behaviors (1969) | 1.00 | 1.46 (1.32, 1.62) | 1.83 (1.66, 2.02) | 2.47 (2.27 2.70) | 2.66 (2.43, 2.91) |
| Low emotional control (1969) | 1.00 | 1.49 (1.34, 1.66) | 1.90 (1.73, 2.10) | 2.60 (2.38, 2.83) | 2.78 (2.55, 3.04) |
| Psychiatric diagnosis (1969) | 1.00 | 1.50 (1.35, 1.66) | 1.90 (1.73, 2.10) | 2.60 (2.39, 2.84) | 2.81 (2.57, 3.06) |
| Musculoskeletal diagnosis (1969) | 1.00 | 1.49 (1.35, 1.66) | 1.91 (1.73, 2.11) | 2.65 (2.43, 2.89) | 2.88 (2.64, 3.14) |
| **Adjusted for all above** | 1.00 | 1.39 (1.25, 1.55) | 1.65 (1.49, 1.83) | 2.03 (1.85, 2.23) | 2.03 (1.84, 2.25) |
| **% reduction of HR** |  | 20% | 28% | 37% | 45% |
| Youth unemployment (1969) | 1.00 | 1.49 (1.34, 1.65) | 1.89 (1.72, 2.09) | 2.59 (2.37, 2.82) | 2.79 (2.56, 3.05) |
| Unemployed in young adulthood (1974-1991) | 1.00 | 1.48 (1.33, 1.64) | 1.90 (1.72, 2.09) | 2.55 (2.34, 2.78) | 2.84 (2.60, 3.10) |
| Unemployed in middle adulthood (1992-2000) | 1.00 | 1.47 (1.32, 1.63) | 1.85 (1.67, 2.04) | 2.47 (2.26, 2.69) | 2.77 (2.53, 3.02) |
| **Adjusted for all unemployment variables** | 1.00 | 1.46 (1.32, 1.62) | 1.84 (1.67, 2.03) | 2.38 (2.18, 2.60) | 2.69 (2.46, 2.94) |
| **% reduction of HR** |  | 6% | 7% | 16% | 10% |
| Long-term sick leave (1990-2000) | 1.00 | 1.40 (1.24, 1.55) | 1.61 (1.46, 1.77) | 2.06 (1.89, 2.25) | 2.20 (2.01, 2.40) |
| Mental diagnosis (1973-2000) | 1.00 | 1.48 (1.33, 1.64) | 1.88 (1.71, 2.08) | 2.54 (2.33, 2.77) | 2.76 (2.53, 3.01) |
| **Adjusted for all health-related variables** | 1.00 | 1.40 (1.26, 1.56) | 1.61 (1.46, 1.55) | 2.03 (1.86, 2.21) | 2.18 (1.99, 2.38) |
| **% reduction of HR** |  | 18% | 33% | 38% | 37% |
| Full model | 1.00 | 1.32 (1.19, 1.47) | 1.44 (1.30, 1.60) | 1.64 (1.50, 1.81) | 1.73 (1.56, 1.90) |
| **% reduction of HR** |  | 34% | 51% | 61% | 61% |
|  |  |  |  |  |  |
| **Number of events (%)** | 642 (9.2) | 794 (13.5) | 1074 (16.8) | 2569 (22.6) | 2231 (24.5) |

SEP: socioeconomic position

Supplementary table 3. Complete case analysis excluding 1908 individuals with missing information on covariates, crude and adjusted hazard ratios (HRs) with 95% confidence intervals (CIs) for the association between educational qualification and non-health-related labor market marginalization among mature-aged workers

| Adjustments | ≥15  HR (95%CI) | 13-14  HR (95%CI) | 12  HR (95%CI) | 10-11  HR (95%CI) | ≤9  HR (95%CI) |
| --- | --- | --- | --- | --- | --- |
| **Crude** | 1.00 | 1.38 (1.20, 1.58) | 1.60 (1.40, 1.83) | 2.05 (1.83, 2.30) | 1.58 (1.40, 1.79) |
| Childhood SEP (1960) | 1.00 | 1.43 (1.25, 1.65) | 1.67 (1.47, 1.91) | 2.18 (1.93, 2.46) | 1.72 (1.52, 1.96) |
| IQ (1969) | 1.00 | 1.35 (1.18, 1.55) | 1.51 (1.32, 1.72) | 1.80 (1.59, 2.04) | 1.31 (1.14, 1.50) |
| Health behaviors (1969) | 1.00 | 1.36 (1.18, 1.56) | 1.55 (1.35, 1.76) | 1.93 (1.72, 2.17) | 1.48 (1.30, 1.68) |
| Low emotional control (1969) | 1.00 | 1.39 (1.21, 1.60) | 1.63 (1.43, 1.86) | 2.04 (1.81, 2.29) | 1.55 (1.37, 1.76) |
| Psychiatric diagnosis (1969) | 1.00 | 1.38 (1.20, 1.58) | 1.60 (1.40, 1.83) | 2.01 (1.79, 2.26) | 1.54 (1.36, 1.74) |
| Musculoskeletal diagnosis (1969) | 1.00 | 1.38 (1.20, 1.58) | 1.60 (1.41, 1.83) | 2.05 (1.83, 2.30) | 1.58 (1.40, 1.79) |
| **Adjusted for all above** | 1.00 | 1.40 (1.22, 1.61) | 1.55 (1.36, 1.78) | 1.84 (1.61, 2.10) | 1.34 (1.17, 1.55) |
| **% reduction of HR** |  | +7% | 8% | 20% | 41% |
| Youth unemployment (1969) | 1.00 | 1.37 (1.19, 1.57) | 1.58 (1.38, 1.80) | 1.95 (1.74, 2.19) | 1.48 (1.31, 1.68) |
| Unemployed in young adulthood (1974-1991) | 1.00 | 1.36 (1.18, 1.56) | 1.59 (1.39, 1.81) | 1.89 (1.68, 2.12) | 1.53 (1.35, 1.73) |
| Unemployed in middle adulthood (1992-2000) | 1.00 | 1.29 (1.08, 1.44) | 1.40 (1.22, 1.59) | 1.55 (1.38, 1.74) | 1.33 (1.17, 1.50) |
| **Adjusted for all unemployment variables** | 1.00 | 1.28 (1.11, 1.47) | 1.39 (1.22, 1.58) | 1.46 (1.29, 1.64) | 1.27 (1.12, 1.44) |
| **% reduction of HR** |  | 27% | 35% | 57% | 53% |
| Long-term sick leave (1990-2000) | 1.00 | 1.35 (1.18, 1.55) | 1.53 (1.34, 1.74) | 1.90 (1.69, 2.14) | 1.46 (1.29, 1.65) |
| Mental diagnosis (1973-2000) | 1.00 | 1.37 (1.19, 1.57) | 1.59 (1.39, 1.81) | 1.98 (1.76, 2.22) | 1.53 (1.35, 1.73) |
| **Adjusted for all health-related variables** | 1.00 | 1.35 (1.18, 1.55) | 1.52 (1.34, 1.74) | 1.86 (1.66, 2.10) | 1.44 (1.27, 1.63) |
| **% reduction of HR** |  | 7% | 13% | 18% | 26% |
| Full model | 1.00 | 1.33 (1.16, 1.53) | 1.40 (1.22, 1.61) | 1.49 (1.31, 1.70) | 1.24 (1.07, 1.43) |
| **% reduction of HR** |  | 15% | 33% | 58% | 59% |
|  |  |  |  |  |  |
| **Number of events (%)** | 376 (5.4) | 435 (7.3) | 541 (8.5) | 1216 (10.7) | 763 (8.4) |

SEP: socioeconomic position

Supplementary table 4: Additional analyses excluding 549 individuals that experienced long-term sickness absence prior to long-term unemployment, crude and adjusted hazard ratios (HRs) with 95% confidence intervals (CIs) for the association between educational qualification and non-health-related labor market marginalization among mature aged workers.

| Adjustments | ≥15  HR (95%CI) | 13-14  HR (95%CI) | 12  HR (95%CI) | 10-11  HR (95%CI) | ≤9  HR (95%CI) |
| --- | --- | --- | --- | --- | --- |
| **Crude** | 1.00 | 1.29 (1.11, 1.48) | 1.45 (1.27, 1.67) | 1.91 (1.69, 2.15) | 1.44 (1.26, 1.63) |
| Childhood SEP (1960) | 1.00 | 1.34 (1.16, 1.55) | 1.52 (1.33, 1.75) | 2.03 (1.80, 2.30) | 1.57 (1.37, 1.79) |
| IQ (1969) | 1.00 | 1.27 (1.10, 1.46) | 1.39 (1.21, 1.60) | 1.73 (1.52, 1.97) | 1.22 (1.06, 1.41) |
| Health behaviours (1969) | 1.00 | 1.27 (1.10, 1.47) | 1.41 (1.23, 1.62) | 1.82 (1.61, 2.05) | 1.36 (1.19, 1.54) |
| Low emotional control (1969) | 1.00 | 1.29 (1.12, 1.49) | 1.45 (1.27, 1.68) | 1.88 (1.67, 2.11) | 1.39 (1.23, 1.58) |
| Psychiatric diagnosis (1969) | 1.00 | 1.29 (1.12, 1.49) | 1.45 (1.27, 1.67) | 1.88 (1.66, 2.11) | 1.40 (1.23, 1.59) |
| Musculoskeletal diagnosis (1969) | 1.00 | 1.29 (1.11, 1.48) | 1.45 (1.27, 1.67) | 1.91 (1.69, 2.15) | 1.44 (1.26, 1.63) |
| **Adjusted for all above** | 1.00 | 1.32 (1.15, 1.53) | 1.44 (1.25, 1.66) | 1.78 (1.56, 2.03) | 1.27 (1.10, 1.47) |
| **% reduction of HR** |  | +14% | 2% | 14% | 38% |
| Youth unemployment (1969) | 1.00 | 1.28 (1.11, 1.47) | 1.43 (1.25, 1.64) | 1.82 (1.61, 2.05) | 1.34 (1.18, 1.53) |
| Unemployed in young adulthood (1974-1991) | 1.00 | 1.27 (1.10, 1.46) | 1.44 (1.26, 1.66) | 1.76 (1.57, 1.99) | 1.39 (1.22, 1.58) |
| Unemployed in middle adulthood (1992-2000) | 1.00 | 1.20 (1.04, 1.39) | 1.26 (1.10, 1.45) | 1.43 (1.27, 1.61) | 1.19 (1.05, 1.36) |
| **Adjusted for all variables related to work history** | 1.00 | 1.19 (1.03, 1.38) | 1.26 (1.10, 1.44) | 1.35 (1.19, 1.52) | 1.15 (1.01, 1.31) |
| **% reduction of HR** |  | 32% | 44% | 62% | 66% |
| Long-term sick leave (1990-2000) | 1.00 | 1.27 (1.10, 1.47) | 1.41 (1.23, 1.62) | 1.83 (1.62, 2.06) | 1.37 (1.20, 1.56) |
| Mental diagnosis (1973-2000) | 1.00 | 1.28 (1.11, 1.47) | 1.44 (1.26, 1.65) | 1.85 (1.64, 2.08) | 1.39 (1.23, 1.58) |
| **Adjusted for all health-related variables** | 1.00 | 1.27 (1.10, 1.46) | 1.41 (1.23, 1.62) | 1.79 (1.59, 2.02) | 1.35 (1.18, 1.53) |
| **% reduction of HR** |  | 6% | 9% | 13% | 20% |
| Full model | 1.00 | 1.27 (1.09, 1.44) | 1.32 (1.16, 1.52) | 1.41 (1.23, 1.61) | 1.18 (1.02, 1.37) |
| **% reduction of HR** |  | 10% | 29% | 55% | 59% |
|  |  |  |  |  |  |
| **Number of events (%)** | 364 (5.0) | 388 (6.4) | 471 (7.2) | 1086 (9.3) | 668 (7.1) |

SEP: socioeconomic position

Supplementary table 5. Competing risk regression for the association between level of education (years) and health-related labor market marginalization among mature aged workers, with death as competing risk.

| Adjustments | ≥15  HR (95%CI) | 13-14  HR (95%CI) | 12  HR (95%CI) | 10-11  HR (95%CI) | ≤9  HR (95%CI) |
| --- | --- | --- | --- | --- | --- |
| **Crude** | 1.00 | 1.51 (1.36, 1.67) | 1.93 (1.75, 2.12) | 2.69 (2.47, 2.93) | 2.89 (2.66, 3.16) |
| Childhood SEP (1960) | 1.00 | 1.47 (1.33, 1.63) | 1.87 (1.70, 2.06) | 2.57 (2.36, 2.80) | 2.74 (2.50, 2.99) |
| IQ (1969) | 1.00 | 1.45 (1.31, 1.60) | 1.75 (1.59, 1.93) | 2.26 (2.07, 2.47) | 2.30 (2.09, 2.52) |
| Health behaviors (1969) | 1.00 | 1.49 (1.34, 1.64) | 1.85 (1.69, 2.04) | 2.52 (2.32, 2.74) | 2.67 (2.46, 2.92) |
| Low emotional control (1969) | 1.00 | 1.52 (1.37, 1.68) | 1.93 (1.76, 2.12) | 2.64 (2.43, 2.88) | 2.79 (2.57, 3.04) |
| Psychiatric diagnosis (1969) | 1.00 | 1.52 (1.37, 1.68) | 1.93 (1.75, 2.12) | 2.65 (2.44, 2.89) | 2.82 (2.58, 3.06) |
| Musculoskeletal diagnosis (1969) | 1.00 | 1.51 (1.37, 1.67) | 1.93 (1.76, 2.13) | 2.69 (2.48, 2.93) | 2.89 (2.65, 3.14) |
| **Adjusted for all above** | 1.00 | 1.42 (1.28, 1.58) | 1.69 (1.53, 1.86) | 2.09 (1.91, 2.30) | 2.07 (1.88, 2.28) |
| **% reduction of HR** |  | 17% | 26% | 35% | 44% |
| Youth unemployment (1969) | 1.00 | 1.50 (1.36, 1.66) | 1.92 (1.74, 2.11) | 2.62 (2.41, 2.85) | 2.79 (2.56, 3.04) |
| Unemployed in young adulthood (1974–1991) | 1.00 | 1.50 (1.35, 1.66) | 1.92 (1.75, 2.11) | 2.59 (2.38, 2.82) | 2.85 (2.62, 3.10) |
| Unemployed in middle adulthood (1992–2000) | 1.00 | 1.49 (1.34, 1.65) | 1.87 (1.70, 2.06) | 2.51 (2.31, 2.73) | 2.78 (2.55, 3.02) |
| **Adjusted for all unemployment variables** | 1.00 | 1.48 (1.34, 1.64) | 1.86 (1.69, 2.05) | 2.42 (2.23, 2.64) | 2.70 (2.47, 2.94) |
| **% reduction of HR** |  | 6% | 7% | 16% | 10% |
| Long-term sick leave (1990-2000) | 1.00 | 1.41 (1.27, 1.56) | 1.62 (1.47, 1.78) | 2.10 (1.93, 2.28) | 2.20 (2.02, 2.40) |
| Mental diagnosis (1973–2000) | 1.00 | 1.50 (1.35, 1.66) | 1.90 (1.73, 2.09) | 2.58 (2.37, 2.81) | 2.77 (2.54, 3.01) |
| **Adjusted for all health-related variables** | 1.00 | 1.41 (1.27, 1.56) | 1.62 (1.47, 1.78) | 2.06 (1.90, 2.25) | 2.19 (2.00, 2.38) |
| **% reduction of HR** |  | 19% | 33% | 37% | 38% |
| Full model | 1.00 | 1.34 (1.21, 1.48) | 1.46 (1.32, 1.61) | 1.69 (1.54, 1.85) | 1.75 (1.59, 1.93) |
| **% reduction of HR** |  | 33% | 50% | 59% | 61% |

SEP: Socioeconomic position

Supplementary table 5. Competing risk regression for the association between level of education (years) and non-health-related labor market marginalization among mature aged workers, with death as competing risk.

| Adjustments | ≥15  HR (95%CI) | 13-14  HR (95%CI) | 12  HR (95%CI) | 10-11  HR (95%CI) | ≤9  HR (95%CI) |
| --- | --- | --- | --- | --- | --- |
| **Crude** | 1.00 | 1.35 (1.18, 1.54) | 1.58 (1.39, 1.79) | 2.05 (1.84, 2.29) | 1.59 (1.41, 1.79) |
| Childhood SEP (1960) | 1.00 | 1.41 (1.23, 1.61) | 1.65 (1.45, 1.88) | 2.18 (1.94, 2.45) | 1.73 (1.53, 1.96) |
| IQ (1969) | 1.00 | 1.32 (1.15, 1.52) | 1.49 (1.31, 1.70) | 1.81 (1.61, 2.05) | 1.32 (1.16, 1.50) |
| Health behaviors (1969) | 1.00 | 1.33 (1.17, 1.53) | 1.52 (1.34, 1.73) | 1.94 (1.73, 2.17) | 1.48 (1.32, 1.68) |
| Low emotional control (1969) | 1.00 | 1.35 (1.18, 1.55) | 1.58 (1.39, 1.79) | 2.02 (1.81, 2.26) | 1.54 (1.37, 1.74) |
| Psychiatric diagnosis (1969) | 1.00 | 1.35 (1.18, 1.55) | 1.58 (1.39, 1.79) | 2.02 (1.81, 2.26) | 1.54 (1.37, 1.74) |
| Musculoskeletal diagnosis (1969) | 1.00 | 1.35 (1.18, 1.54) | 1.58 (1.39, 1.79) | 2.05 (1.84, 2.29) | 1.59 (1.41, 1.79) |
| **Adjusted for all above** | 1.00 | 1.38 (1.20, 1.58) | 1.54 (1.35, 1.76) | 1.86 (1.64, 2.11) | 1.36 (1.18, 1.56) |
| **% reduction of HR** |  | +9% | 7% | 18% | 39% |
| Youth unemployment (1969) | 1.00 | 1.34 (1.17, 1.53) | 1.55 (1.37, 1.77) | 1.96 (1.74, 2.18) | 1.48 (1.31, 1.67) |
| Unemployed in young adulthood (1974–1991) | 1.00 | 1.33 (1.16, 1.52) | 1.56 (1.38, 1.78) | 1.90 (1.69, 2.12) | 1.53 (1.36, 1.73) |
| Unemployed in middle adulthood (1992–2000) | 1.00 | 1.26 (1.10, 1.44) | 1.37 (1.21, 1.56) | 1.55 (1.38, 1.74) | 1.33 (1.18, 1.50) |
| **Adjusted for all unemployment variables** | 1.00 | 1.25 (1.10, 1.43) | 1.36 (1.20, 1.55) | 1.45 (1.30, 1.63) | 1.27 (1.13, 1.44) |
| **% reduction of HR** |  | 27% | 36% | 56% | 53% |
| Long-term sick leave (1990-2000) | 1.00 | 1.32 (1.16, 1.51) | 1.51 (1.32, 1.71) | 1.91 (1.71, 2.14) | 1.47 (1.30, 1.66) |
| Mental diagnosis (1973-2000) | 1.00 | 1.34 (1.17, 1.53) | 1.56 (1.37, 1.77) | 1.98 (1.77, 2.21) | 1.53 (1.36, 1.73) |
| **Adjusted for all health-related variables** | 1.00 | 1.32 (1.16, 1.51) | 1.50 (1.32, 1.71) | 1.87 (1.67, 2.10) | 1.45 (1.28, 1.63) |
| **% reduction of HR** |  | 8% | 13% | 17% | 25% |
| Full model | 1.00 | 1.30 (1.14, 1.49) | 1.39 (1.21, 1.59) | 1.45 (1.27, 1.65) | 1.25 (1.08, 1.43) |
| **% reduction of HR** |  | 13% | 33% | 57% | 58% |

SEP: Socioeconomic position
